# Updates on Clinical and Genetic Heterogeneity of *ASPM* in 12 Autosomal Recessive Primary Microcephaly Families in Pakistani Population

**DOI:** 10.3389/fped.2021.695133

**Published:** 2021-07-06

**Authors:** Niaz Muhammad Khan, Basharat Hussain, Chenqing Zheng, Ayaz Khan, Muhammad Shareef Masoud, Qingquan Gu, Linhui Qiu, Naveed Altaf Malik, Muhammad Qasim, Muhammad Tariq, Junlei Chang

**Affiliations:** ^1^Department of Bioinformatics and Biotechnology, Government College University Faisalabad, Faisalabad, Pakistan; ^2^Shenzhen Key Laboratory of Biomimetic Materials and Cellular Immunomodulation, Institute of Biomedicine and Biotechnology, Shenzhen Institute of Advanced Technology, Chinese Academy of Sciences, Shenzhen, China; ^3^University of Chinese Academy of Sciences, Beijing, China; ^4^Shenzhen Real Omics Biotech Co., Ltd., Shenzhen, China; ^5^National Institute for Biotechnology and Genetic Engineering (NIBGE-C), Faisalabad, Pakistan; Pakistan Institute of Engineering and Applied Sciences (PIEAS), Islamabad, Pakistan

**Keywords:** primary microcephaly, MCPH5, whole exome sequencing, Pakistani population, founder effect

## Abstract

Microcephaly (MCPH) is a genetically heterogeneous disorder characterized by non-progressive intellectual disability, small head circumference, and small brain size compared with the age- and sex-matched population. MCPH manifests as an isolated condition or part of another clinical syndrome; so far, 25 genes have been linked with MCPH. Many of these genes are reported in Pakistani population, but due to a high rate of consanguinity, a significant proportion of MCPH cohort is yet to be explored. MCPH5 is the most frequently reported type, accounting for up to 68.75% alone in a genetically constrained population like Pakistan. In the current study, whole exome sequencing (WES) was performed on probands from 10 families sampled from South Waziristan and two families from rural areas of the Pakistani Punjab. Candidate variants were validated through Sanger sequencing in all available family members. Variant filtering and *in silico* analysis identified three known mutations in *ASPM*, a MCPH5-associated gene. The founder mutation p.Trp1326^*^ was segregating in 10 families, which further confirmed the evidence that it is the most prominent mutation in Pashtun ethnicity living in Pakistan and Afghanistan. Furthermore, the previously known mutations p.Arg3244^*^ and p.Arg1019^*^ were inherited in two families with Punjab ethnic profile. Collectively, this study added 12 more families to the mutational paradigm of *ASPM* and expanded the Pakistani MCPH cohort.

## Introduction

Microcephaly (MCPH, OMIM#251200) is a neurodevelopmental disorder characterized by a small head circumference, non-progressive intellectual disability, and small brain size compared with the age- and sex-matched population. The prevalence of MCPH5, the most frequently reported type, is 1/10,000 in consanguineous populations, such as Pakistan's; sporadically, its probability is 1/1,000,000 in non-consanguineous European population (1, 2). Occipitofrontal circumference (OFC) ranges between −2 and −8 SD (standard deviation) at birth (32 and 26 cm, respectively) (3). Patients may exhibit mild to severe developmental delay, sloping forehead, epilepsy, and hereditary hearing loss. Predominantly, primary microcephaly shows an autosomal recessive mode of inheritance (4, 5). So far, mutations in 25 genes have been linked with MCPH. These genes mainly express during cell division and mutations lead to disruption in neurogenesis, cell cycle checkpoints, and centrosome and spindle positioning. The result is an architecturally normal brain with reduced volume, especially in the cerebral cortex region (6). Most of these genes were implicated in the last 10 years after the advent of cutting-edge sequencing technologies (7). Since ~85% of the known variants for rare genetic diseases occur in the coding subset of DNA, therefore, exome sequencing is a handy approach for quick genetic diagnosis, thereby reducing the number of variants for follow-up studies (8, 9). Exome sequencing has the advantage of sequencing the entire coding subset of the genome in a single experiment.

Biallelic mutations in *ASPM* (MCPH5 MIM #605481) occur more frequently than other genes in MCPH, underlying up to 40% of all the reported primary microcephaly cases. *ASPM* (MIM# 605481), located on chromosome 1q31.3, is 6.2 kb long with 28 exons, coding for 3,477 amino acids (Ensemble, GRCh38/hg38). This gene plays a vital role in the division of neural progenitor cells and in controlling the cell cycle by helping symmetric proliferative division, as well as asymmetric neurogenic divisions. *ASPM* knockdown in animal models leads to a decrease in cortical area and microcephaly as observed in humans (10, 11).

In the current study, 12 unrelated families were investigated. Of these, 10 families, with multiple patients, belong to the Wazir tribe of Pashtuns, located in the remote district of South Waziristan. The other two families are from rural areas of Pakistani Punjab and are mutually unrelated. Genetic analysis of these families revealed three known mutations in *ASPM*, a MCPH5-associated gene. In 10 Wazir families the variant c.3978G>A (p.Trp1326^*^) was segregating, whereas the two families from Punjab had two different *ASPM* variants c.9730C>T (p.Arg3244^*^) and c.3055C>T (p.Arg1019^*^). This study, thus, further endorses *ASPM* mutations as the most frequent cause of microcephaly. The p.Trp1326^*^ variant has been previously reported as a founder mutation in more than 50 families among various Pashtun tribes including Wazir (4, 12). The segregation of this variant in 10 unrelated Wazir families in the current study suggests that this variant represents an old mutation and that mutations in this gene are a rare event.

## Materials and Methods

### Study Participants and Pedigree Construction

Patients were recruited according to the diagnostic criteria of reduced head circumference (HC) (≥3 SD), intellectual disability, and absence of brain malformation symptoms. These families were ascertained through field surveys and with the help of local healthcare workers. All the patients in participating families were characterized with primary microcephaly. Written informed consent was obtained from the guardians of patients and relevant family members. Pedigrees were drawn using HaploPainter v.2.0 (GPLv2) (13). Eighteen patients from the 12 families were male (53%), whereas 16 were female (47%). Ages of the patients range from 1 month to 40 years (Table 1). In seven of these families, patients were born to consanguineous parents. In the remaining five families, there was no immediate consanguinity; however, parents of the patients belong to same tribes (Supplementary Figure 1). HC was measured and compared with a graph of age- and sex-dependent HC values of the normal population (14). Peripheral blood samples were collected from 34 available affected individuals and their normal sibling in EDTA tubes. For molecular analysis, genomic DNA was extracted from blood leukocytes according to standard protocol (15). The Institutional Review Board of the Government College University Faisalabad (Pakistan) and Shenzhen Institute of Advanced Technology, Chinese Academy of Sciences approved the study.

**Table 1:** Clinical and genetic manifestations in the MCPH cohort.

**Family individual ID**		**Mutation**	**Affected member**	**Age at last assessment** **(years)**	**Head circumference** **(cm)/(SD)**	**Ethnicity**	**Intellectual disability**	**Epilepsy**	**Hearing** **loss**	**Skeletal anomalies**	**Ophthalmological anomalies**
Family 1	Gender	c.3978G>A (p.Trp1326^*^)	5			Wazir (Pashtun)					
IV:2	M			3	42/−4.5		+	+	+	Club feet, protruding ears	–
IV:3	F			18	42/−11.6		+	+	+	+	–
IV:5	F			26	43/−10.7		–	+	+	–	–
Family 2			5			Wazir (Pashtun)					
III:2	M			Deceased	ND		+	+	+	–	–
III:4	M			14	36/−11.7		+	+	+	–	Strabismus
III:5	M			26	39/−10.8		+	+	+	–	+
III:6	F			24	39/−14.4		+	+	+	–	–
III:7	F			Deceased	ND		+	+	+	–	+
Family 3			4			Wazir (Pashtun)					
II:3	M			Deceased	ND		+	+	+	Bedridden	–
II:5	M			40	43/−8.2		+	+	+	Bedridden	–
II:7	F			Deceased	ND		+	+	+		–
II:8	F			Deceased	ND		+	+	+		–
Family 4			5			Wazir (Pashtun)					
IV:1	M			Deceased	ND		+	+	+	Club feet	Strabismus
IV:2	M			Deceased	ND		+	+	+	Club feet	Strabismus
IV:3	M			19	40/−10.2		+	+	+	Club feet	Cataract
IV:10	F			11	38/−11.9		+	+	+	+	Strabismus
IV:11	F			15	41/−11.8		+	+	+	+	Cataract
Family 5			2			Wazir (Pashtun)					
IV:1	M			10	33/−15.5		+	+	–	–	Strabismus
IV:3	M			16	35/−13.5		+	+	–	–	+
Family 6			3			Wazir (Pashtun)					
III:2	M			7	43/−6.8		–	–	–	–	–
III:4	M			2	40/−6.1		–	–	–	–	–
Family 7		c.3978G>A	10			Wazir (Pashtun)					
		(p.Trp1326^*^)									
IV:5	M			Deceased	ND		+	+	–	Bedridden	Strabismus
IV:7	M			27	39/−10.8		+	+	–	–	+
IV:8	F			Deceased	ND		+	+	–	Bedridden	+
IV:10	F			23	38/−15.4		+	+	–	–	+
V:4	F			6	37/−11.3		+	+	–	–	+
V:7	M			Deceased	ND		+	+	–	–	+
V:8	M			21	46/−6.1		+	+	–	–	+
V:10	F			13	38/−12.2		+	+	–	–	+
V:13	M			24	39/−10.8		+	+	–	–	+
V:16	F			10	42/−8.0		+	+	–	–	+
Family 8			1			Wazir (Pashtun)					
V:1	F			2	33/−10.2		+	+	–	Protruding ears	–
Family 9			2			Wazir (Pashtun)					
III:1	M			8	46/−4.6		–	–	–	Bedridden	–
III:3	F			10	33/−15.1		–	–	–	+	+
Family 10			3			Wazir (Pashtun)					
IV:5	F			18	37/−16.3		+	+	+	–	Strabismus
IV:6	F			2	33/−10.2		+	+	+	–	–
IV:7	F			1 month	ND		+	–	+	–	–
Family 11		c.9730C>T	3			Punjabi					
		(p.Arg3244^*^)									
IV:3	M			35	42/−8.8		–	–	–	–	–
IV:4	M			42	44/−7.5		+	–	–	–	–
IV:5	F			Deceased	ND		–	–	–	–	–
Family 12		c.3055C>T	3			Punjabi					
		(p.Arg1019^*^)									
III:2	M			25	45/−6.8		+	–	+	–	–
IV:3	M			19	41/−9.5		+	+	+	–	–
IV:5	F			13	39/−11.4		+	+	+	–	–

*M, male; F, female; +, feature is present; – feature is missing; ND, not determined; SD, standard deviation; cm, centimeter*.

### Whole Exome Sequencing

DNA sample from a proband in each family was selected for whole exome sequencing (WES), using Illumina NovaSeq 6000 platform. Agilent V6 array was used to capture exons with ~100× depth of coverage, using paired-end 2 × 150 bp protocol (2021 Illumina, Inc., San Diego, CA, USA). Sequenced data were aligned and mapped to the human reference genome sequence (GRCh37) assembly. Variants were called using the Genome Analysis Tool Kit GATK, version 3.7 (16), and all variants were annotated and classified by utilizing SnpEff (version 4.2; http://snpeff.sourceforge.net/). After annotation, variants were filtered in public databases [1,000 Genomes Project and Genome Aggregation Database (gnomAD)] and those with MAF <0.005 were retained. Among these variants, homozygous and compound heterozygous variants were selected because the most likely inheritance pattern for these pedigrees was autosomal recessive. No further prediction tools were used as all the variants are known to result in premature termination of translation.

### Sanger Sequencing

Candidate variants from exome sequencing were validated by bidirectional Sanger sequencing each variant in the proband. Validated variants were then bidirectionally Sanger sequenced in all the available family members for segregation analysis. Sanger sequencing was done using BigDye terminator sequencing chemistry in Genetic Analyzer 3730 (Applied Biosystems, Foster City, CA, USA). Sequence chromatograms were analyzed with sequence analysis software DNASTAR (Lasergene, Madison, WI, USA) and Sequencher 5.4.6 (Gene Codes Corporation, Ann Arbor, MI, USA).

## Results

### Clinical and Molecular Findings of MCPH Families

This study reports on three previously known *ASPM* variants in 12 Pakistani MCPH families with 34 affected individuals (18 males and 16 females). Clinical investigation of all affected individuals revealed reduced skull size (head circumference below −5 SD), mild to severe intellectual disability, slopping forehead, and protruding ears. In addition, speech impairment, ophthalmological problems, deafness, and skeletal defects were noted in the affected individuals (Supplementary Figures 1, 2 and Table 1).

**Figure 1:**
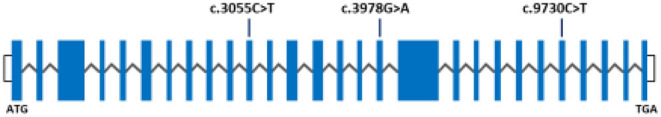
Schematic representation of the *ASPM* gene on 1q31, showing exons (blue rectangles) and introns (black zigzag lines). White blocks represent the location of the untranslated regions. *ASPM* is the main driver of primary microcephaly. The previously known mutations c.3055C>T, c.3978G>A, and c.9730C>T were found in this study.

WES was performed on the proband in each family. The resulting FASTQ files were transformed to BAM, and the BAM files were converted to variant call format (vcf) file. The resultant variants were utilized for the identification of the variant that may lead to the disease based on low frequency (MAF +0.01%). We applied various filters and bioinformatics tools to further narrow down the candidate variants and confirmed them in the large reference population cohort of the gnomAD (*n* > 120,000 exomes and >150,000 genomes). Variants that survived various bioinformatics filters/tools (as explained above) were analyzed, and the previously reported non-sense variants in *ASPM* gene were found in all the families under investigation. Mutations c.3978G>A (p.Trp1326^*^), c.9730C>T (p.Arg3244^*^), and c.3055C>T (p.Arg1019^*^) were found in homozygous form (Figure 1; Supplementary Figure 1). The sequences of oligos for Sanger confirmation are available in Supplementary Table 1.

## Discussion

We investigated 12 Pakistani families with primary microcephaly by WES. Sanger validation of the candidate variants and segregation analysis found a non-sense *ASPM* variant c.3978G>A (p.Trp1326^*^) in 10 of these families; the remaining two families had two different non-sense *ASPM* variants segregated, c.9730C>T (p.Arg3244^*^) and c.3055C>T (p.Arg1019^*^). The parents and some of the siblings of the homozygous patients are heterozygous carriers for these *ASPM* variants, whereas other siblings are homozygous for the wild-type allele, which corresponds to the recessive inheritance pattern of MCPH in these families. *ASPM* mutations result in abnormal spindle-like microcephaly-associated protein, which is responsible for 40 to 68% of MCPH incidence (17, 18).

The first variant c.3978G>A has been previously reported in only one Punjabi family and 49 Pashtun families, including three families from the Wazir tribes (4). A large cohort of non-Pashtun Pakistani families with MCPH did not carry this variant (6). The variant was, therefore, regarded as a founder mutation in Pashtun tribes of Pakistan and Afghanistan (4). With the 10 Wazir families included in this study, the total number of Pashtun families with this segregated variant rose to 59. Thus, our finding further endorses this variant as a founder mutation in various tribes of Pashtuns. Screening for this single variant alone can help in identifying a significant number of carriers in any prospective screening program.

The remaining two families were found to have two more *ASPM* variants, c.9730C>T, (p.Arg3244^*^) and c.3055C>T (p.Arg1019^*^). These variants have also been previously reported (19) and characterized. Our findings support a considerable clinical variability associated with *ASPM* mutations, and we do not see any clear-cut genotype–phenotype correlations in the patients. In conclusion, in 12 families from Pakistan, we found three known mutations in the well-characterized *ASPM* gene. The high frequency (10 out of 10 Pashtun families) of the *ASPM* mutation p.Trp1326^*^ among Pashtun families hints on its role as a founder mutation among Pashtuns, especially the Wazir tribe, of Pakistan and Afghanistan. Therefore, testing for this mutation, among Pashtuns, as a first step will be a cost-effective and time-saving approach in the future while performing genetic analysis of MCPH families from this and other populations (20). Similarly, testing for this mutation can also help in population-wide screening for carrier screening and prenatal diagnosis to prevent further affected births.

## Data Availability Statement

The sequencing data analyzed in this study is not readily accessible due to privacy restrictions with the patients. Requests to access these datasets should be directed to the corresponding author MQ ( qasimawan@gcuf.edu.pk).

## Ethics Statement

This study was approved from the ethical committee of Government College University, Faisalabad, Pakistan and Shenzhen Institute of Advanced Technology, Chinese Academy of Sciences. Written informed consent to participate in this study was provided by the participants' legal guardian/next of kin. Written informed consent was obtained from the individual(s), and minor(s)' legal guardian/next of kin, for the publication of any potentially identifiable images or data included in this article.

## Author Contributions

NK, BH, and CZ recruited the patients, gathered detailed clinical information for the study, and wrote the initial draft. MQ, JC, and MT designed the study. AK, MM, and NM critically reviewed and edited the manuscript. LQ and QG performed whole exome evaluation and mutational analyses. MQ and JC directed the project. All authors critically reviewed the paper. All authors contributed to the article and approved the submitted version.

## Conflict of Interest

CZ and QG were employed by the company Shenzhen Real Omics Biotech Co., Ltd. The remaining authors declare that the research was conducted in the absence of any commercial or financial relationships that could be construed as a potential conflict of interest.
