# Supplementary material for: Updates on Clinical and Genetic Heterogeneity of ASPM in 12 Autosomal Recessive Primary Microcephaly Families in Pakistani Population
Source: Front Pediatr. 2021 Jul 6;9:695133. doi: 10.3389/fped.2021.695133 (PMC8290066; doi:10.3389/fped.2021.695133)
Supplement: Supplementary file 1 [file Data_Sheet_1.PDF]

Supplementary data

Family I

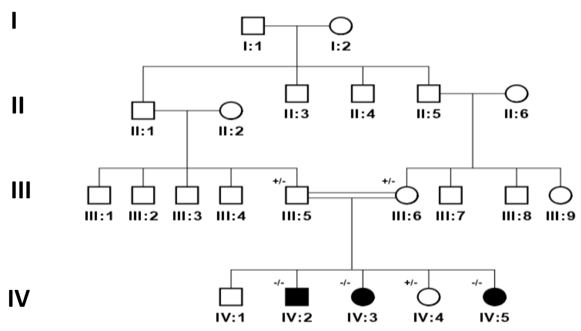

Family II

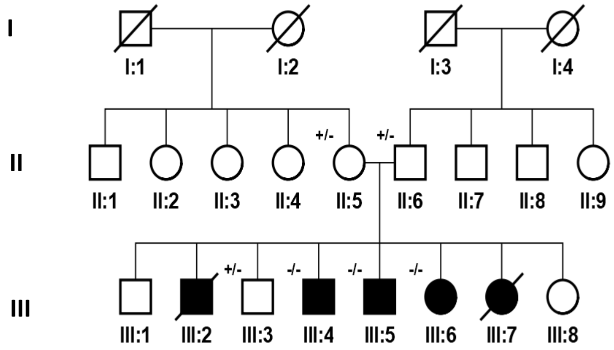

Family III

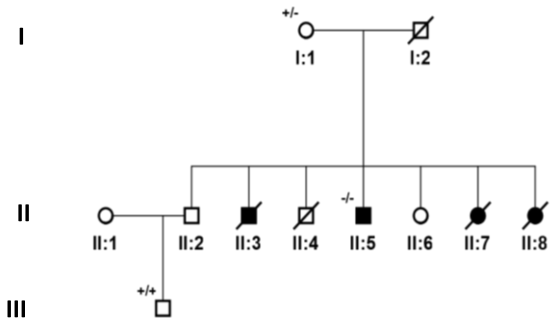

Family IV

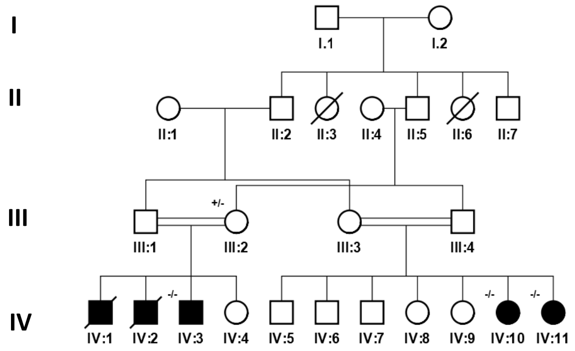

Family V

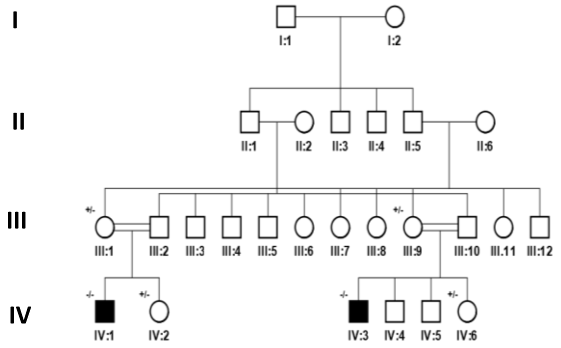

Family VI

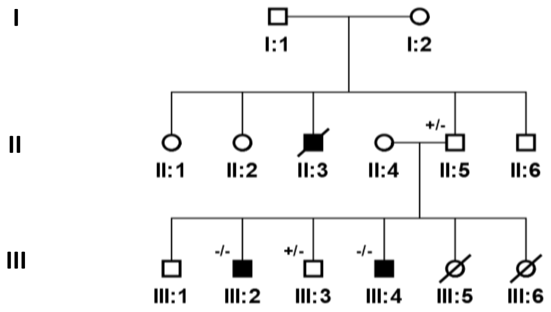

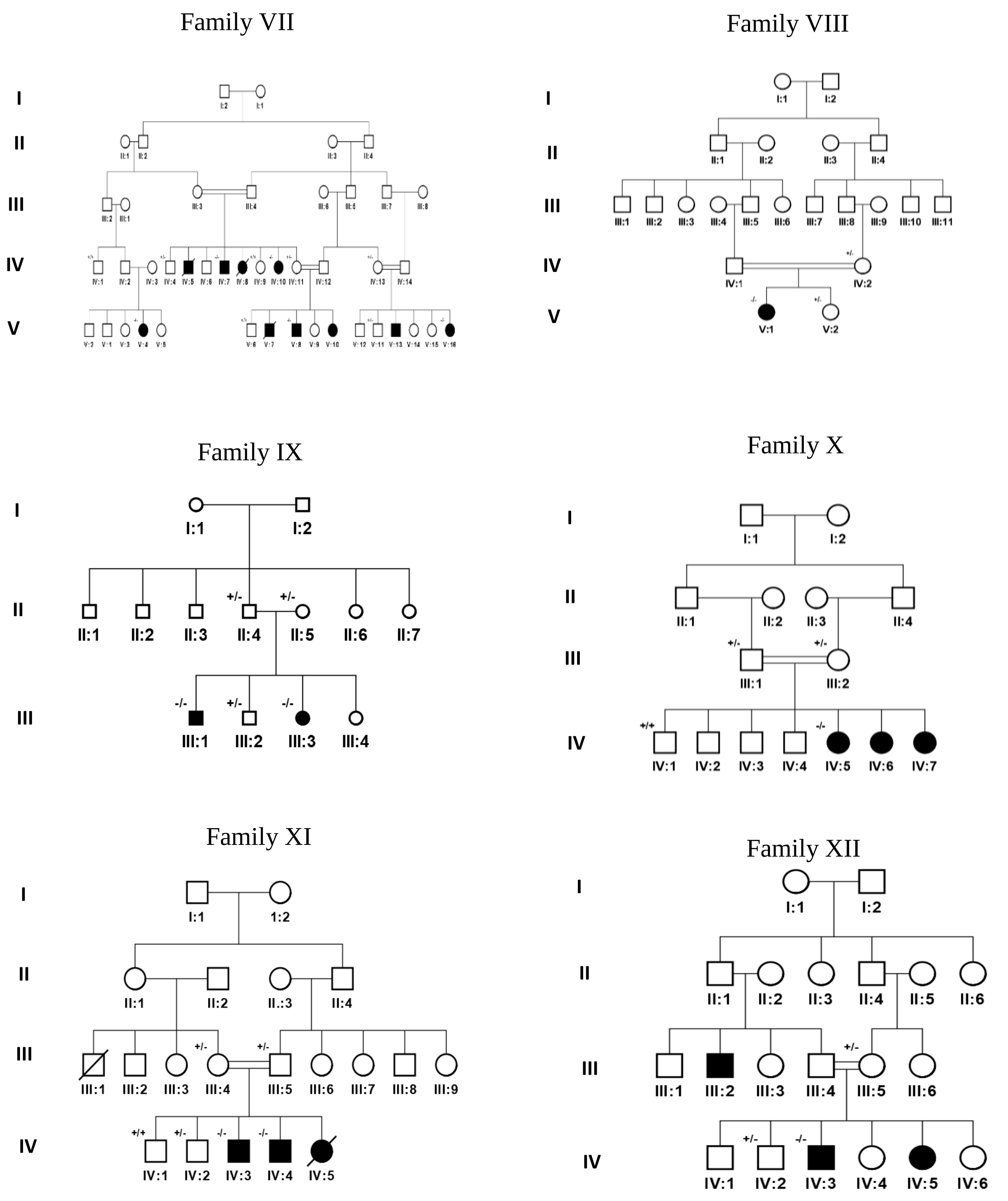

Supplementary figure 1. Pedigrees of 12 MCPH families harboring *ASPM* mutations, participated in the study. Unfilled circles/squares represent unaffected female/male respectively. Dark filled indicates affected member in the pedigree and squares and circles with diagonal line represent the deceased individuals. In each family, one affected was subjected to whole exome sequencing. Variants were validated by Sanger sequencing in all available individuals.

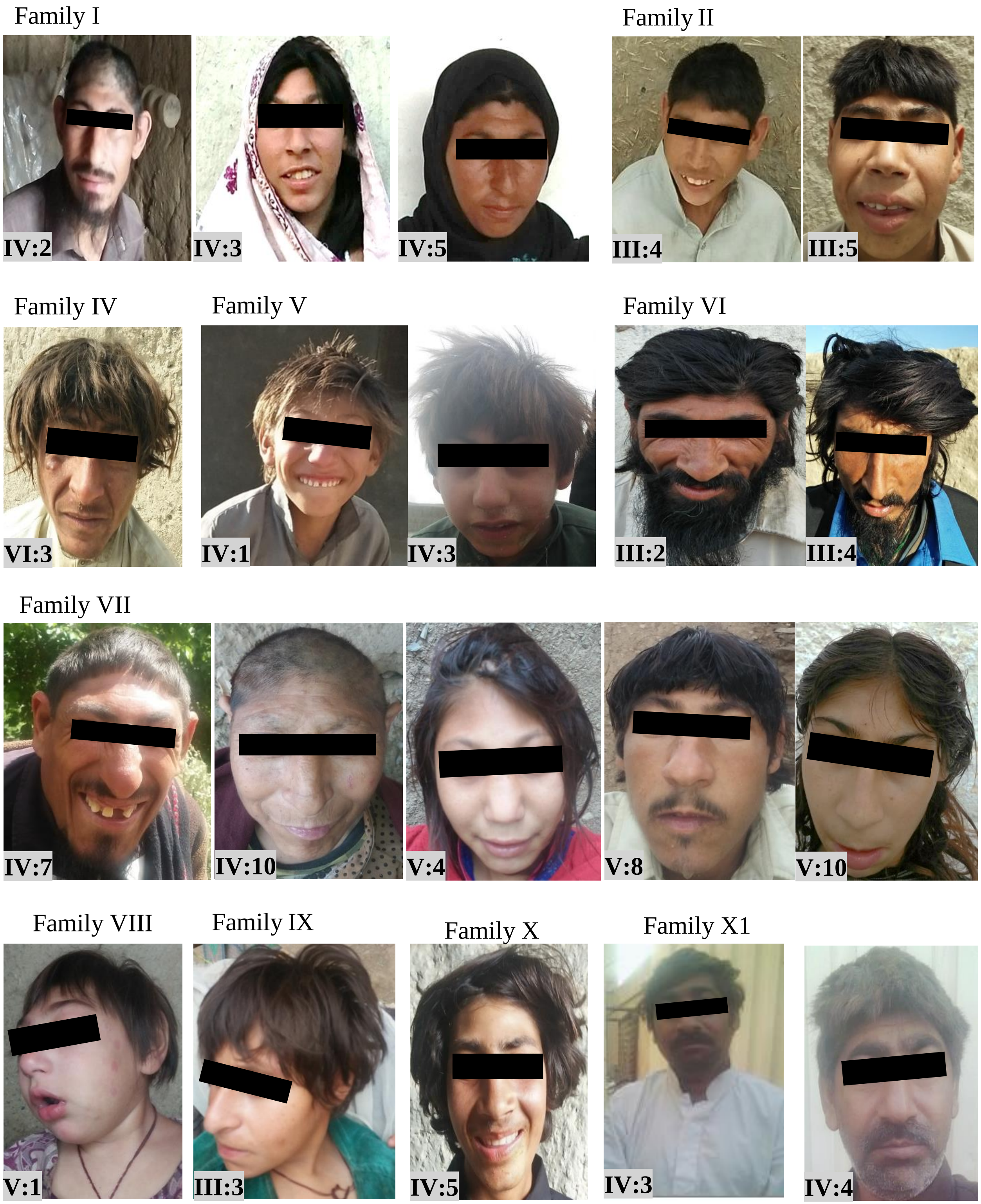

Supplementary Figure 2. Photographs and clinical features of MCPH families participated in the current study. Family 3 and 12 were not willing to use their pictures for publication.

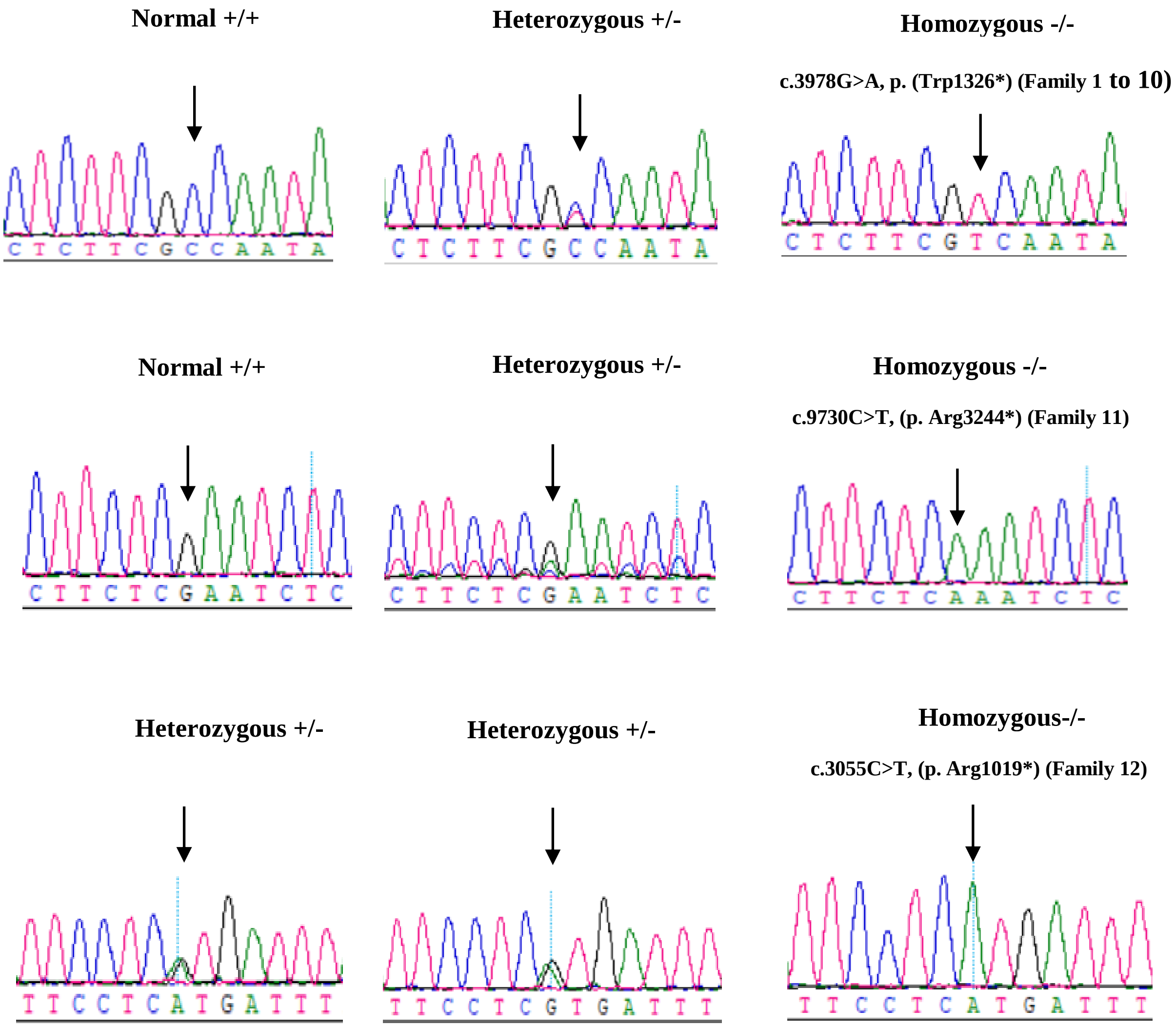

Supplementary figure 3. Chromatograms showing ASPM mutations p. (Trp1326*) in 10 families having Pashtun ethnicity, p. (Arg3244*) and p. (Arg1019*) segregate in two families with Punjab ethnic background. All these are previously known mutations from Pakistan and other population with high rate of inbreeding.

Supplementary Table S1: Sequence of oligos used for amplification of ASPM mutations		Mutation
**ID**	**Sequence 5' - 3'**	
GCMCPH32-FOR	TAAAGCCCTGTAACTGTTGG	c.3055C>T
GCMCPH32-REV	CTGTGCCTATCCACAATATC	
GCMCPH18-FOR	GAAGAGAGCAACAGGTACAG	c.3978G>A
GCMCPH18-REV	GAGTTAATGCAGCACTCGTC	
GCMCPH24-FOR	TTACAGCAACAAAATGAAGT	c.9730C>T
GCMCPH24-REV	TACTGCGATTACAACTTCGG	
